# Analysis of Social Media Discussions on (#)Diet by Blue, Red, and Swing States in the U.S.

**DOI:** 10.3390/healthcare9050518

**Published:** 2021-04-29

**Authors:** Amir Karami, Alicia A. Dahl, George Shaw, Sruthi Puthan Valappil, Gabrielle Turner-McGrievy, Hadi Kharrazi, Parisa Bozorgi

**Affiliations:** 1 School of Information Science, University of South Carolina, Columbia, SC 29208, USA; 2 Department of Public Health Sciences, University of North Carolina at Charlotte, Charlotte, NC 28223, USA; adahl3@uncc.edu (A.A.D.); gshaw11@uncc.edu (G.S.J.); 3 Computer Science and Engineering Department, University of South Carolina, Columbia, SC 29208, USA; sruthi@email.sc.edu; 4 Arnold School of Public Health, University of South Carolina, Columbia, SC 29208, USA; mcgrievy@mailbox.sc.edu (G.T.-M.); bozorgip@email.sc.edu (P.B.); 5 Bloomberg School of Public Health, Johns Hopkins University, Baltimore, MD 21205, USA; kharrazi@jhu.edu; 6 South Carolina Department of Health and Environmental Control, Columbia, SC 29201, USA

**Keywords:** politics, diet, social media, health, text mining

## Abstract

The relationship between political affiliations and diet-related discussions on social media has not been studied on a population level. This study used a cost- and -time effective framework to leverage, aggregate, and analyze data from social media. This paper enhances our understanding of diet-related discussions with respect to political orientations in U.S. states. This mixed methods study used computational methods to collect tweets containing “diet” or “#diet” shared in a year, identified tweets posted by U.S. Twitter users, disclosed topics of tweets, and compared democratic, republican, and swing states based on the weight of topics. A qualitative method was employed to code topics. We found 32 unique topics extracted from more than 800,000 tweets, including a wide range of themes, such as diet types and chronic conditions. Based on the comparative analysis of the topic weights, our results revealed a significant difference between democratic, republican, and swing states. The largest difference was detected between swing and democratic states, and the smallest difference was identified between swing and republican states. Our study provides initial insight on the association of potential political leanings with health (e.g., dietary behaviors). Our results show diet discussions differ depending on the political orientation of the state in which Twitter users reside. Understanding the correlation of dietary preferences based on political orientation can help develop targeted and effective health promotion, communication, and policymaking strategies.

## 1. Introduction

Eating is an important social activity and an expression of local cultures and beliefs [1]. A poor diet is a significant contributing factor to the leading causes of chronic diseases in the United States [2]. Adults who follow a healthy diet live longer and have a lower risk of obesity, heart disease, type 2 diabetes, and cancer [3]; however, most Americans do not maintain healthy diets [3]. Annually, $147 billion is spent on health programs for obesity [3] and more than 70% of U.S. adults are overweight or obese [4].

Political behavior, such as voting, can be considered a social determinant of health [5,6,7,8,9]. To detect and track patterns of individual behaviors (e.g., diet and political behavior) on a population level, social monitoring was proposed as the first step [10]. For example, two large social monitoring studies found that democrats or unaffiliated individuals are more likely to follow a vegan or vegetarian-based diet compared to republicans [11,12]. Another diet and political behavior study found that republicans reported eating more high fat and processed foods and were less likely to eat fruits and vegetables and/or participate in exercise [7].

Political and health behavior studies employ surveys to collect data; however, surveys are expensive, labor-intensive, and usually based on small or medium sample sizes [13]. Additionally, surveys are often limited to the topics covered by their underlying questions; hence, limiting the understanding of complex, multi-faceted population level health communication factors, strategies, and policies [13]. Consequently, public health experts are increasingly considering new sources of health and social information to collect, analyze, and monitor larger population samples [14,15].

Social media sites have become mainstream channels of communication, with growing popularity across the U.S. in the last decade [16]. In 2019, 72% of U.S. adults used at least one social media site that was readily available on mobile devices [17]. Social media platforms have also provided opportunities for people to share their health opinions, concerns, and experiences, thus offering potentially new information for public health use [10]. For example, the political orientation of locations (e.g., U.S. states) can be linked to social media data to explore and analyze politically-driven diet-related trends and concerns [18]. Such analytical solutions can improve our understanding of common topics and concerns expressed by individuals residing in states with different political orientations; thus, leading public health officials/policymakers to develop more efficient health communication strategies.

Recent social media studies have analyzed food consumption [1,19] and diet-related discussions [20] of users on a population level. Additionally, social media studies have utilized correlation analysis to investigate the relationship between food-related tweets and rates of obesity and diabetes across geographies [21], and between unhealthy-related tweets and disadvantaged areas [22]. Nonetheless, research is lacking in examining social media diet-related discussions based on a state’s political orientation of the users’ geographical locations. This study proposes an effective framework to leverage and aggregate data from social media and to use secondary data analysis to enhance our understanding of diet-related discussions with respect to the political orientations of U.S. states. This research can help identify health behavior patterns, understand differences, tailor social media posts for promotion and communication purposes, and develop public health policies based on political orientations. This study investigates whether a significant difference exists between diet-related discussions on social media in democratic, republican, and swing states.

## 2. Materials and Methods

**Figure 1:**
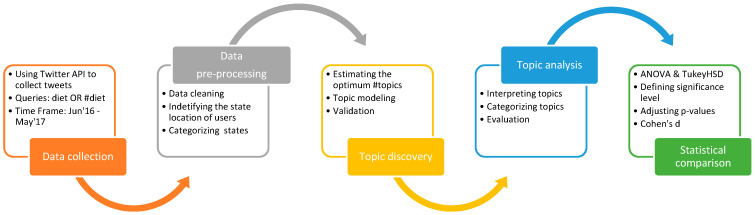
Research Framework.

This research involved multiple steps, including data collection, data pre-processing, topic discovery, topic analysis, and statistical comparison (Figure 1). We utilized both quantitative and qualitative methods to achieve the study analysis.

### 2.1. Data Collection

Twitter has more than 320 million users, generating 500 million tweets per day [23]. In 2019, the number of monthly active U.S. Twitter users reached 68 million [24]. Twitter users can share their messages (tweets) or repost tweets of other users (retweets). To collect data, we utilized twitter4j [8], a Twitter API developed in the Java platform. We collected tweets containing “diet” or “#diet” posted between June 2016 and May 2017. This process also provided the state location of users for determining the political orientation of the state.

### 2.2. Data Pre-Processing

First, we developed methods to remove retweets starting with RT, hashtags starting with #, and usernames starting with @. Second, we obtained the location of users from their profiles to find U.S. users and their state names. Third, we determined whether the location of a user belonged to a democratic (blue), republican (red), or swing state. We used the election analytics website FiveThirtyEight [25] to identify 12 swing states that regularly saw close contests over the last few presidential campaigns. Sixteen and 22 states regularly voted for democrat and republican candidates, respectively [25]. Individual-level political orientations of Twitter users were not available for this study, hence, similar to a past study conducted by Hswen et al. [26], we focused on the political environment of Twitter users based on their geographical associations.

### 2.3. Topic Discovery

Next, we identified emerging topics discussed in the collected tweets using topic modeling. Among topic models, latent Dirichlet allocation (LDA) [27] is a popular topic model that has been applied on short-length documents (e.g., tweets) as well as long-length documents (e.g., research papers) [28]. LDA is a valid and widely used model for detecting themes in a corpus [28]. LDA is a generative probabilistic model providing two matrices: P(word|topic) and P(topic|document). The earlier matrix recognizes semantically related words representing a theme. For example, applying LDA on our corpus provided a topic representing “diet pill” with the following words: “*weight,” “diet,” “fat,” “pill,”* and *“belly”.* The P(topic|document) matrix showed the distribution of topics for a document (tweet), which assisted in finding tweets most related to a topic. LDA was used for different applications, such as politics [29], opinion mining [30], and social media analysis [31,32,33]. LDA was also utilized for analysis of health comments on social media, such as characterizing diet, diabetes, obesity, exercise [18], COVID-19 discussions [34], and LGBT health [35]. However, we could not locate research, using LDA, to investigate health-related social comments based on the political orientation of each state.

The output of LDA for *n* documents (tweets), *m* words, and *t* topics were two matrices. The first one was the probability of each of the words for each topic or *P*(*W_i_|T_k_*) and the second one was the probability of each of the topics for each document or *P*(*T_k_|D_j_*):
Topics
Documents$Words\left[\begin{array}{ccc} P(W_{1}|T_{1}) & \cdots & P(W_{1}|T_{t})\\ \vdots & \ddots & \vdots \\ P(W_{m}|T_{1}) & \cdots & P(W_{m}|T_{t}) \end{array}\right]$&$Topics\;\left[\begin{array}{ccc} P(T_{1}|D_{1}) & \cdots & P(T_{t}|D_{n})\\ \vdots & \ddots & \vdots \\ P(T_{t}|D_{1}) & \cdots & P(T_{t}|D_{n}) \end{array}\right]$*P*(*W_i_*|*T_k_*)
*P*(*T_k_*|*D_j_*)

The top words in each topic based on the order of *P*(*W_i_|T_k_*) represent the topic. To calculate the average weight of a topic per tweet (A_WT), we used the following formula. For example, A_WT(T_x_) > A_WT(T_y_) means that topic x was discussed more than topic *y.*

$$A\_WT\left(T_{k}\right)=\frac{\sum_{j=1}^{n}P(T_{k}|D_{j})}{n}$$

**Figure 2:**
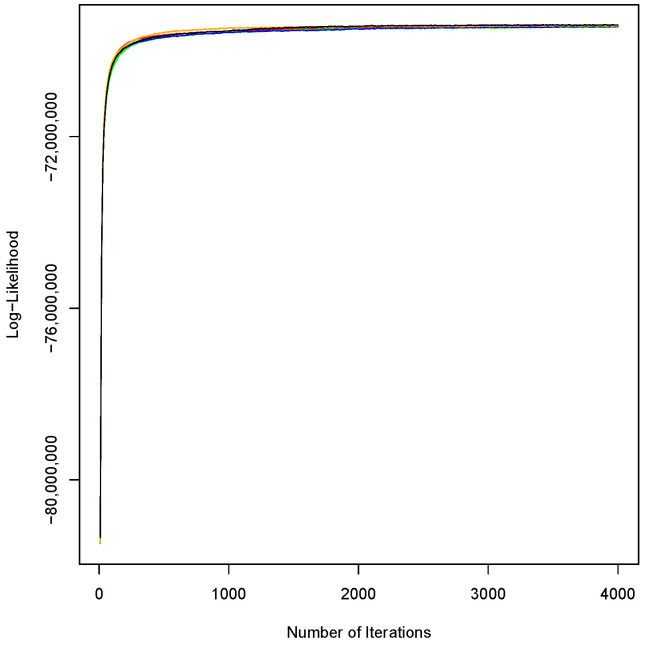
Convergence of the log-likelihood for five sets of 4000 integrations.

Before applying LDA, we needed to estimate the number of topics based on the level of consistency and coherence of topics. We utilized the C_V method, which is highly correlated with human ratings [36], developed in the gensim Python package [37], to measure the coherence for the number of topics from 2 to 100 topics. This step offered the optimum number of topics at 41. To find topics, we utilized the Mallet implementation of LDA [38]. We set the number of topics and iterations at 41 and 4000, respectively. We also used the list of stop words in Mallet to remove most common words, such as “a” and “the.” To assess the robustness of LDA, this study compared five sets of 4000 iterations and found no significant difference between the mean and standard deviation of the log-likelihood of the sets (Figure 2).

### 2.4. Topic Analysis

To code the identified topics, we applied a qualitative approach with the following phases to disclose the meaning of topics and their categories:

**Phase 1.** Identify meaningful and health-related topics. To interpret the topics, two of the authors coded the topics individually by reading the top words (shown in Table 1) and top tweets for each of the topics using P(*T_k_*|*D_j_*). In this phase, the coders answered two questions for each topic. The first question (Q1) was “Does the topic have a meaningful theme?” If the answer of Q1 was positive, the second question (Q2) was “Does the topic contain a health-related issue?” This phase filtered out the topics that were not meaningful or not related to health.

**Phase 2.** Label creation of topics. The two coders used consensus coding [39] to create a label (theme) for each of topics. In this phase, the coders addressed a third question (Q3), “What is a proper label to represent the topic?” For consensus coding, the coders first developed labels separately. To have standard labels, the coders met, described their labels, and compared and contrasted the labels they had each generated. They could change or keep their initial labels.

**Phase 3.** Categorizing topics. The coders used the consensus coding to develop categories. Then, coders assigned topics to those categories independently. The weight of categories for each tweet is measured using the summation of the weight of topics in a category. For example, if two topics were in a category and the weight of the topics were 0.2 and 0.1 for a tweet, the weight of category for the tweet would be 0.3. A third coder resolved the disagreements between the two coders in phases 2 and 3.

To develop the topic categories identified in Table 2, we utilized prior literature covering this research domain [18,40,41]. Moreover, one of the coders’ research on health behavior change strategies and nutrition and physical activity promotion provided the expertise to develop the representative categories based on the aforementioned domain knowledge. Significant deliberation was required to establish the chosen categories that were subsequently applied to the identified health topics. The developed topic categories were then applied and further analyzed (Q3) in Phase 4.

**Phase 4.** Assessing the reliability of coding. To find the agreement between the coders, the agreement percentage was used to determine the amount of data that were erroneous. Agreements between both programs were consistent; the results were incorporated into our analyses. The agreements were 85.4% for Q1, 87.8% for Q2, 87.7% for Q3, and 89.5% for phase 3. Additionally, Cohen’s kappa was performed to determine if there was agreement between the two coders’ due to uncertainty resulting from random chance. According to a study examining inter-rater reliability [40], we found a fair level of agreement for Q1 (k = 0.32), and substantial levels of agreement for Q2 and Q3, respectively (k = 0.92 and 0.88).

### 2.5. Statistical Comparison

We developed statistical tests to compare democratic, republican, and swing states based on the mean of the weight of topics. We applied an analysis of variance (ANOVA), which tested whether the weight of topics was different for democratic, republican, and swing states, developed in the *aov* function of the R stats package [41]. We used the weight of topics as the dependent variable. After we found that the means of the democratic, republican, and swing states differed, we utilized Tukey’s multiple comparison test [42] to find which of the means were different significantly. We applied the TukeyHSD function developed in the R stats package [41]. When there is a large sample size, the level of significance level should be set at a lower level [43]. We defined the passing *p*-value at 0.0005 based on our sample size using $\frac{0.05}{\sqrt{\frac{N}{100}}}$ [44], where *N* is the number of tweets. To control familywise errors of multiple ANOVAs, we used the false discovery rate (FDR) method [45] developed in the p.adjust function of the R stats package [41]. FDR, compared to Bonferroni correction, reduces not only false positives, but also false negatives [46]. This step helped us address the following question: are the differences in the average weight of topics among democratic, republican, and swing states significant?

To identify the magnitude of the differences, we used the absolute effect size using Cohen’s d calculated by dividing the mean difference by the pooled standard deviation [47]. The original Cohen’s d index was classified as small (d = 0.2), medium (d = 0.5), and large (0.8) effect sizes [48]. The Cohen’s d index classification was also extended to include very small (d = 0.01), small (d = 0.2), medium (d = 0.5), large (d = 0.8), very large (d = 1.2), and huge (d = 2.0) effect sizes [49]. However, the Cohen’s d classification has two limitations. First, the classification is based on small sample sizes [48]. Second, the average effect size in large samples is less than small samples [50]. For example, most effect size values in a study with more than 14,000 data points are found to be in or below the small threshold [51]. To address the limitations of applying Cohen’s d on large datasets, we measured the mean of the effect sizes of sample sizes used in developing the initial Cohen’s d classification [48], including 8, 40, 60, 100, 200, 500, and 1000 random tweets, instead of all tweets analyzed in the study.

## 3. Results

We collected 33,049,693 million tweets. The pre-processing step provided 1,009,169 tweets posted by U.S. users. Among these tweets, 151,892 tweets were posted by U.S. users who did not mention their state in their profile. Out of 875,277 tweets containing state information, most tweets were posted in democratic states (58%) followed by republican (24%) and swing (18%) states.

**Figure 3:**
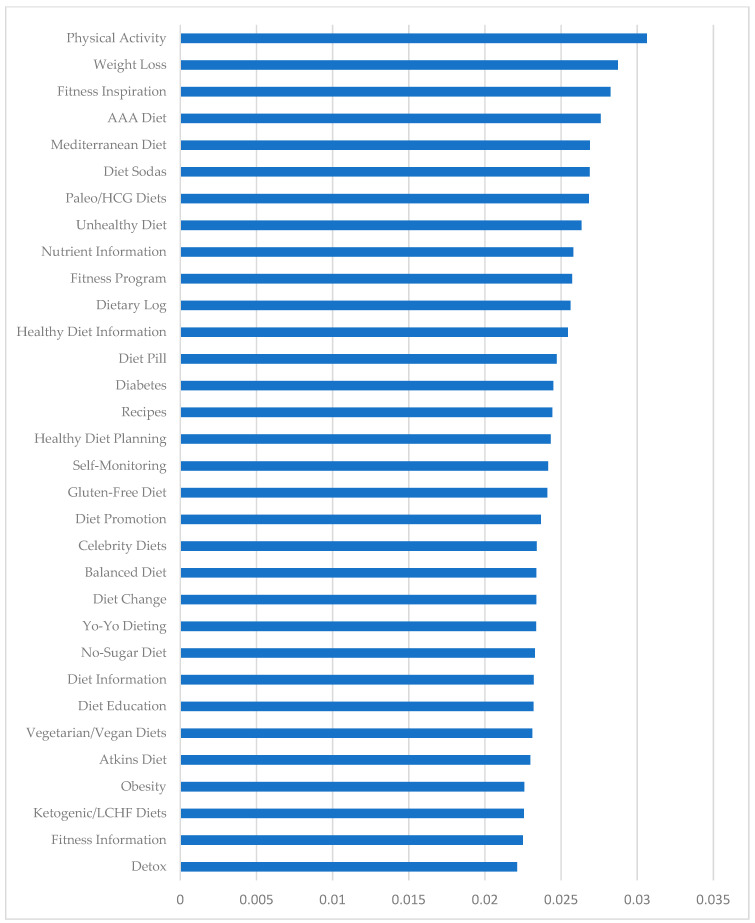
Average weight of topics per tweet.

**Figure 4:**
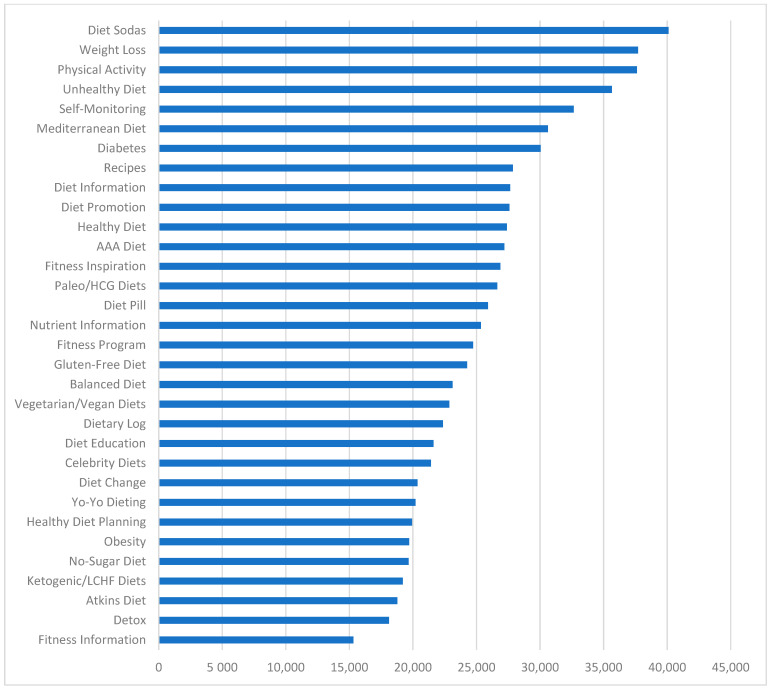
The number of predominant tweets in each topic.

From the 41 topics provided by LDA, we found 32 meaningful and relevant topics. These topics covered a range of matters, such as self-monitoring and different types of diets (Table 1). We offered a definition for each of the topics, based on reviewing the top words per topic in Table A1, top tweets per topic using P(T|D), and related content on the web. Figure 3 shows the average weight of each topic per tweet from the highest frequency topic (i.e., physical activity) to the lowest frequency topic (i.e., detox). Figure 4 shows the number of tweets predominantly in each topic. For example, the highest and the lowest number of tweets were related to diet sodas and fitness information, respectively. We used the Kendall and Spearman tests to compare the ranking of topics in Figure 3 and Figure 4. Both tests showed a significant (*p*-value = 0.000 < 0.05) positive moderate to strong correlation (tau = 0.6 and rho = 0.77) between the ranking of Figure 3 and the ranking of Figure 4 [52].

After topic discovery and analysis, we examined the difference between democratic, republican, and swing states based on the average weight of the 32 topics. The results in Table 2 show a significant difference between democratic, republican, and swing states based on the average weight of 32 topics. Our findings in Table 2 show:

- No significant difference between republican and swing states across 10 topics, including self-monitoring, diet sodas, recipes, celebrity diets, nutrition information, healthy diet planning, AAA diet, fitness inspiration, fitness information, and dietary log. However, a significant difference was detected between republican and swing states based on 22 (68.75%) topics, in which swing states had higher discussion than republican states on 13 topics.
- No significant difference between democratic and republican states in five topics, including diet information, diet pills, vegetarian/vegan, diet change, and Atkins diets. However, a significant difference was identified between republican and democratic states in 27 (84%) topics, in which republican states had higher discussion than democratic states on 17 topics.
- No significant difference between democratic and swing states on the diabetes topic. However, a difference was detected between democratic and swing states in 31 (96.875%) topics, in which swing states had higher discussion than democratic states in 22 topics.
- While the republican and swings states discussed the types of diets more than other states, the discussions of democratic states focused on positive and negative outcomes of diets, such as weight loss and chronic diseases. Compared to the republican and the swing states, the democratic states made more mentions of fitness role models (e.g., celebrities) to inspire healthy behaviors.
- U.S. news scored and ranked diets based on their healthiness, in which the higher score represented a healthier diet [53]. Based on this ranking, the democratic states were more interested in diets with a higher healthy score representing the value of a diet for improving health and helping fight diseases, such as the Mediterranean (4.8/5) and AAA (3/5) diets; however, the republican and swing states were more interested in diets with a lower healthy score, such as paleo (2.5/5), and ketogenic/LCHF (Keto) (1.7/5). Studies also show that Yo-Yo [54] and gluten-free diets for people without celiac disease [55] are not healthy diets.

These findings show that the maximum difference was between democratic and swing states, and the minimum difference was between republican and swing states. Out of 96 comparisons (32 topics × 3 types of states), we found 80 significant differences, indicating 83.33% dissimilarity between democratic, republican, and swing states.

Table 3 shows the average Cohen’s d values representing the effect size of differences between the sample sizes regarding each comparison. As shown in the table, out of the 80 significant differences, 30 had very small (d = 0.01) and 50 had small (d = 0.2) effect sizes, indicating that the differences were not trivial on a population level.

Based on the average weight of each topic per each tweet, Table 4 shows the top-10 topics for democratic (D), republican (R), and swing (S) states, illustrating their preferences. In Table 4, the class label shows whether a topic is among top-10 topics of D, R, or S states. For example, class D illustrates that a topic is among top-10 topics of democratic states but is not among top-10 topics of republican and swing states. Mediterranean diet, diet sodas, unhealthy diet, and fitness program (class DRS) were common in the top-10 list for democratic, republican, and swing states. The highest (80%) overlap was between republican and swing states (RS), and the lowest one (40%) was between democratic and republican/swing states. Physical activity, AAA diet, fitness inspiration, weight loss, nutrient information, and diabetes were topics covered by democratic states (class D) only. In addition, balanced and gluten-free diet topics, and diet promotion and information topics appeared uniquely with republican and swing states (class RS), respectively.

To provide a better insight to the differences between social media users in democratic, republican, and swing states, the coders assigned the topics to the following categories: (1) behavior and lifestyle, (2) health information, (3) chronic conditions, and (4) type of diet (Table 5).

Category 1: behavior and lifestyle. This category encompasses actions taken related to diet, health, and wellbeing, and shows the evidence of patterned behaviors for weight loss. Within the behavior and lifestyle category, topics include self-monitoring, diet soda, unhealthy diet, dietary log, diet promotion, physical activity, healthy diet planning, fitness inspiration, and fitness program. Self-monitoring health behaviors have been shown to provide a sense of accountability and success in weight control trials [56]. Social media users may be tweeting about their personal diet behaviors as a way to self-monitor. Additionally, social media provides a digital space for esteem-support [57,58]. Social networking groups designed specifically for content related to diets may help explain a user’s willingness to share their diet behaviors. Furthermore, research supports adherence to a healthy lifestyle, which includes a combination of multiple health behaviors such as diet and exercise that can reduce one’s mortality risk [59]. Taken together, these healthy behaviors practiced over time can become a lifestyle, and in the case of diet and exercise, have positive health benefits. In general, the democratic and republican states had a higher discussion than the swing states in the first category (Table 6).

Category 2: health information. This category includes sources of advice, details, general information, and promotions about healthy lifestyles. An example of a health information tweet is, “extreme dieting doesn’t belong in an exercise regimen but eating healthily lets you push harder.” The prevalence of topics related to health information across political states supports Twitter as a social media platform to collect health information. This evidence highlights the opportunity it presents for public health and other health agencies and companies to disseminate health information [60]. Moreover, the current divisive political climate across states concerning health guidelines supports the need for a broad impact of health behavior research [7]. Twitter is an effective platform for this information dissemination [61]. For example, considering the weight loss topic, a user wrote, “officially lost pounds in the last ten days since changing my diet and beginning to exercise again, woo-hoo!!” In general, the democratic states had the highest discussion of the second category, followed by republican states, then swing states (Table 6).

Category 3: chronic condition. This category focuses on a disease or health condition that is persistent or long lasting; it also focuses on the bodily effects or experiences of a diet. It is not surprising that diabetes is commonly discussed in relation to diet. As a behavioral modification, a healthy diet is often used as a prescriptive and preventative measure to reduce the effects of or prevent the onset and severity of such disease [62]. For instance, the Mediterranean diet is associated with a decreased risk of colon and breast cancer [63,64]. The data indicate that the third category was discussed more across democratic and swing states, compared to republican states (Table 6).

Category 4: types of diet. This category refers to a specific diet for weight loss or health purposes, as well as general references to food or nutritional components. The fourth category was more popular for the republican and swing states than the democratic states (Table 6).

Table 7 shows the average of Cohen’s d values and effect sizes of sample sizes regarding the categories. This table illustrates that, out of the 10 significant differences, there were five very small (d = 0.01) and five small (d = 0.2) effect sizes, indicating that the differences were not trivial on a population level.

**Figure 5:**
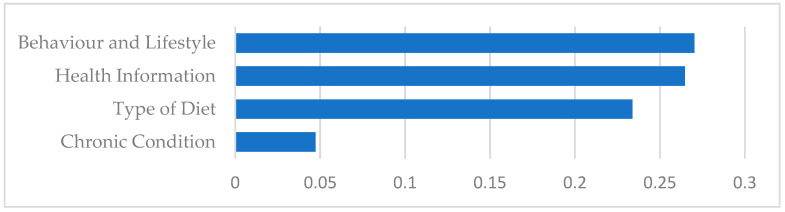
Average weight of categories per tweet.

As depicted in Figure 5, the ranking of the average weight of categories per tweet from the highest to the lowest is (1) behavior and lifestyle, (2) health information, (3) type of diet, and (4) chronic condition. While health information contains more topics than other categories, the average weight of behavior and lifestyle category are more than other categories, indicating that the first category covers popular topics in our corpus.

## 4. Discussion

Social media information can be used efficiently to inform public health agencies on emerging population-level trends and concerns. Political behavior, a growing and dividing social determinant of health, is not well studied regarding health behaviors and outcomes on a population level. Past studies have explored the use of Twitter data to identify diet trends; however, political affiliation and consequent health behaviors have not been studied within that context. This study provides a new approach in studying the association of political behavior with different dietary topics and behaviors on a national level. This research is the first to provide empirical insights into using social media data to understand population-level discussions around diets regarding the political environment of users.

Our findings may provide new insights and complementary information to public health experts and policy makers when developing customized interventions and/or diet-related policies. Our research proposes a new perspective to consider the political orientation of locations when studying diet discussions on social media. Consequently, our work aligns with prior studies that found, when compared to democrats, republicans ate fewer vegetables and fruits while consumed more high fat and processed (unhealthy) foods [7,65], were less interested in searching for health information [7], had lower odds of exercise participation [7], and considered self-monitoring more than liberals (democrats) [66]. According to the literature, democrats are more likely to consume a vegan or vegetarian-based diet [11] and vegan is a popular term in democratic states [21]. While there is no significant difference between democratic and republican states based on the average weight of the vegetarian/vegan, a significant difference exists between based on the average weight of the Mediterranean topic. The foundation of the Mediterranean diet is vegetables, fruits, herbs, nuts, beans and whole grains [67]. While there is a lack of health research on studying swing states, our work addresses this limitation by investigating diet-related discussions in swing states.

Moreover, our findings indicate democratic states discuss chronic illness more, while republican states tweet more often about different diets. A potential explanation to consider is that the public health priorities of the respective states may broadly guide social media conversation topics through health promotion efforts. According to the CDC’s Chronic Disease Prevention [68], 9 of the 22 republican states (40.9%) have a top 5 public health priority specific to obesity or diet, while only 5 of the 16 democratic states (31.3%) emphasize obesity or diet.

Our framework has been applied to diet-related tweets; however, this framework is generalizable for other public health issues. Our study not only offers an efficient and timely approach to explore health-related comments on a population level, but also provides new directions for investigating politics and common health issues on Twitter. The application of this work can be used on other social media sites and health communication platforms aimed at developing efficient health communication and policymaking strategies. While this study specifically focused on Twitter, our approach can be applied to other social media platforms, such as Instagram and Facebook.

Social determinants of health are increasingly being evaluated in health service outcomes studies and are used to bridge the gap between population and public health informatics efforts [69]. Despite the growing role of social and behavioral data in population health management [70], political affiliation has not been studied on a population level to inform public health outcomes. Our study offers a new perspective on potential political association on health choices, such as dietary behaviors. Our results show that diet discussions differ regarding the political orientation of the state in which someone resides. Additional studies are needed to investigate both political environment and various health outcomes using new sources of population-level data, such as social media.

This research shows that a wide range of topics are posted on Twitter, which have different priorities for users in democratic, republican, and swing states. In fact, our findings have valuable cues that assist in identifying preferences of users in different states. Our work encourages agencies to consider political orientation of U.S. states in a more data-driven approach. There is a need to develop time- and cost-effective methods to access appropriate data. In contract to studies using analog observation, we leveraged naturalistic observation [71] by analyzing public available text data on Twitter, which can complement surveys and studies focusing on analyzing social determinants of health.

Our findings and approach may be useful in monitoring changes in dietary social media posts, identifying areas discussing information, behavior, and lifestyle related to unhealthy diets and chronic conditions, considering political orientation factors in formulating dietary policies, and developing hypotheses to study each identified topic, along with the political orientation of locations. Political orientation is unlikely to have a causal link to health, but we agree that it could be used for health-promotion [72]. Broadly, we believe that social media could be seen as a new data sensor in dietary research using geopolitical dimensions to link diet and politics.

Although this research provides new insights into using social media data to understand population-level discussions around diet, it has some notable limitations. (1) Twitter is an elective service used by 22% of U.S. adults who are mostly between 18 and 49 years old, are more likely to identify as democrats, have a high income, and are more educated than the general public [73]. (2) We focused on the state level geographies that do not necessarily represent the political orientation of the smaller spatial units (e.g., county). (3) We lacked additional demographic information (e.g., race and gender) of the users in this research. (4) We excluded the users who did not share their location in their Twitter profiles. (5) Our data collection was limited to two queries of publicly available tweets, indicating that we might miss other possible relevant data. (6) Our analysis included the tweets posted for one year, but the detected patterns might change across multiple years. (7) Our results cannot be interpreted as causal but our correlational results can be invested as new hypotheses in future work. (8) While what people eat may be commonly discussed online, our approach may not be applicable to other more personal health issues, such as HIV and sexual health, which are often not included in public Tweets. (9) Twitter users often post negative comments and might be interested to post tweets regarding unhealthy foods when they are in a fast-food restaurant [22]. (10) While the categories of topics in Table 5 are based on prior literature, to our knowledge, a gold standard approach for categorizing dietary themes are lacking. Thus, another study may apply a different set of categories. Despite this limitation, our work provides a baseline approach for conducting such analysis. Finally, social media research results are always limited to data generated by people using a specific platform, such as Twitter, within a specific timeframe. Hence, our results should not be generalized to all residents in a geographical area or implied to represent long-term trends in the broader population. Despite these limitations, however, the novel approach and the results of our study can provide researchers with real-time insight and preliminary findings needed to hypothesize and conduct elaborate population-level dietary and political studies.

## 5. Conclusions

To improve monitoring diet patterns in the U.S., this research provides an efficient approach using both computational and qualitative methods to compare democratic, republican, and swing states based on the weight of topics of diet-related discussions on Twitter. We found significant differences among democratic, republican, and swing states, with different preferences based on diet-related topics on Twitter. Our findings can help public health professionals and policymakers target their messaging by developing policies to inform and encourage healthy diet-related behaviors based on local political orientation.

Future research could address the limitations of this research by analyzing the political orientation based on smaller location units (e.g., county, urban, and rural), utilizing methods such as advanced Twitter geolocating techniques [74], and utilizing more queries, expanding the time period to more than one year. While the focus of this study is on the political orientation of U.S. states, future work can develop customized machine learning classifiers to identify the political affiliations of users and transfer the analysis of this research to a user level study.

## Figures and Tables

**Table 1:** A total of 41 topics extracted from the tweets by the LDA methodology.

Label	Top Words per Topic
Self-Monitoring	diet, pounds, days, lost, weeks, months, month, past, year, ago
Weight Loss	weight, loss, diet, program, weightloss, tips, plan, workout, fast, healthy
Diet Information	diet, healthy, tips, balanced, eat, maintain, care, strong, body, essential
Diabetes	diet, diabetes, blood, type, high, pressure, improve, pain, stress, reduce
Diet Sodas	diet, coke, drink, pepsi, soda, caffeine, drpepper, tastes, drank, cherry
Diet Promotion	diet, work, bad, exercise, people, good, body, word, fad, matter
Diet Pill	weight, diet, fat, pill, belly, fast, burn, weightloss, garcinia, appetite
Unhealthy Diet	diet, eat, pizza, ice, cream, lunch, dinner, donuts, candy, cookies
Gluten-Free Diet	diet, recipes, paleo, gluten-free, special, cookbook, food, delicious, healthy, mediterranean
Vegetarian/Vegan Diets	diet, vegan, vegetarian, food, make, eat, plantbased, parents, organic, meat
Diet Education	diet, video, plan, day, meal, paleo, guide, playlist, book, ketogenic
Recipes	diet, chicken, cheese, salad, recipe, soup, rice, fries, pizza, pasta
Balanced Diet	diet, balanced, chocolate, protein, cream, ice, milk, cake, cookie, snack
Paleo/HCG Diets	diet, week, weight, plan, lose, paleo, hcg, day, meal, menu
Healthy Diet	diet, healthy, foods, fruits, daily, veggies, great, vegetables, fiber, superfoods
Physical Activity	weightloss, fitness, diet, health, gym, workout, fatloss, gymtime, yoga, bodybuilding
Mediterranean Diet	diet, risk, cancer, mediterranean, heart, disease, reduce, diabetes, prevent, brain
Detox	diet, detox, water, day, tea, green, body, juice, cleanse, drink
Ketogenic/LCHF Diets	diet, ketogenic, based, keto, plant, great, lchf, lifestyle, food, lowcarb
Obesity	diet, gut, health, brain, obesity, metabolism, immune, microbiome, bacteria, dna
Celebrity Diets	diet, workout, plan, routine, secrets, celebrity, reveals, kardashian, body, kim
Diet Change	diet, change, big, food, people, make, health, poor, mental, habits
Nutrient Information	health, wellness, nutrition, weightloss, diet, foods, natural, vitamin, supplement, lowcarb
No-Sugar Diet	diet, soda, sugar, cut, drink, bad, water, cutting, regular, stop
Atkins Diet	diet, atkins, low, carb, fat, high, protein, calorie, fiber, cholesterol
Healthy Diet Planning	diet, food, healthy, eat, nutrition, make, lifestyle, healthier, live, tips
Yo-Yo Dieting	dieting, eating, extension, yo-yo, tips, make, good, eat, avoid, loseweight
AAA Diet	fitness, weightloss, health, diet, fatloss, aaadiet, tips, burnfat, natural, loseweight
Fitness Inspiration	weight, diet, healthy, exercise, fatloss, fitspiration, tips, solution, weightloss, nutrition
Fitness Program	diet, start, today, day, tomorrow, back, week, gonna, working, strict, ready, month, gym
Fitness Information	diet, fitness, goals, workingout, leanmuscle, common, mistakes, biggest, myths, success
Dietary Log	eat, diet, feel, cut, meat, good, dairy, thing, food, made

**Table 2:** Comparison of democratic (Dem), republican (Rep), and swing states. Not significant (NS): adjusted *p*-value > 0.0005, significant (*): adjusted *p*-value ≤ 0.0005.

Topic	ANOVA	Tukey Multiple Comparison Test		
	F-Value	Rep vs. Swing	Rep vs. Dem	Dem vs. Swing
Self-Monitoring	457.6 *	NS	* Rep > Dem	* Dem < Swing
Weight Loss	2155.6 *	* Rep > Swing	* Rep < Dem	* Dem > Swing
Diet Information	161.3 *	* Rep < Swing	NS	* Dem < Swing
Diabetes	28.6 *	* Rep < Swing	* Rep < Dem	NS
Diet Sodas	1433.7 *	NS	* Rep > Dem	* Dem < Swing
Diet Promotion	392.3 *	* Rep < Swing	* Rep > Dem	* Dem < Swing
Diet Pill	68.4 *	* Rep > Swing	NS	* Dem > Swing
Unhealthy Diet	1342.9 *	* Rep > Swing	* Rep > Dem	* Dem < Swing
Gluten Free Diet	742.5 *	* Rep > Swing	* Rep > Dem	* Dem < Swing
Vegetarian/Vegan Diets	36.1 *	* Rep < Swing	NS	* Dem < Swing
Diet Education	89.8 *	* Rep < Swing	* Rep > Dem	* Dem < Swing
Recipes	111.8 *	NS	* Rep > Dem	* Dem < Swing
Balanced Diet	1402.6 *	* Rep > Swing	* Rep > Dem	* Dem < Swing
Paleo/HCG Diets	430.3 *	* Rep > Swing	* Rep > Dem	* Dem < Swing
Healthy Diet Information	405.2 *	* Rep < Swing	* Rep > Dem	* Dem < Swing
Physical Activity	9349.9 *	* Rep > Swing	* Rep < Dem	* Dem > Swing
Mediterranean Diet	81.1 *	* Rep < Swing	* Rep < Dem	* Dem < Swing
Detox	349.8 *	* Rep > Swing	* Rep > Dem	* Dem < Swing
Ketogenic/LCHF Diets	292.4 *	* Rep < Swing	* Rep > Dem	* Dem < Swing
Obesity	79.1 *	* Rep < Swing	* Rep < Dem	* Dem > Swing
Celebrity Diets	45.7 *	NS	* Rep < Dem	* Dem > Swing
Diet Change	29.4 *	* Rep < Swing	NS	* Dem < Swing
Nutrient Information	2629.8 *	NS	* Rep < Dem	* Dem > Swing
No-Sugar Diet	461.1 *	* Rep < Swing	* Rep > Dem	* Dem < Swing
Atkins Diet	35.6 *	* Rep < Swing	NS	* Dem < Swing
Healthy Diet Planning	104.8 *	NS	* Rep > Dem	* Dem < Swing
Yo-Yo Dieting	183.6 *	* Rep < Swing	* Rep > Dem	* Dem < Swing
AAA Diet	9386.1 *	NS	* Rep < Dem	* Dem > Swing
Fitness Inspiration	4788.5 *	NS	* Rep < Dem	* Dem > Swing
Fitness Program	879 *	* Rep > Swing	* Rep > Dem	* Dem < Swing
Fitness Information	72.1 *	NS	* Rep < Dem	* Dem > Swing
Dietary Log	954 *	NS	* Rep > Dem	* Dem < Swing

**Table 3:** Cohen’s d and effect size of comparisons in Table 2.

Topic	Mean of Cohen’s d of Sample Sizes			Effect Size		
	Rep vs. Swing	Rep vs. Dem	Dem vs. Swing	Rep vs. Swing	Rep vs. Dem	Dem vs. Swing
Self-Monitoring	NS	0.1	0.2	NS	Very Small	Small
Weight Loss	0.2	0.2	0.2	Small	Small	Small
Diet Information	0.1	NS	0.1	Very Small	NS	Very Small
Diabetes	0.1	0.1	NS	Very Small	Very Small	NS
Diet Sodas	NS	0.1	0.2	NS	Very Small	Small
Diet Promotion	0.2	0.1	0.1	Small	Very Small	Very Small
Diet Pill	0.1	NS	0.2	Very Small	NS	Small
Unhealthy Diet	0.1	0.2	0.2	Very Small	Small	Small
Gluten Free Diet	0.1	0.2	0.1	Very Small	Small	Very Small
Vegetarian/Vegan Diets	0.1	NS	0.2	Very Small	NS	Small
Diet Education	0.2	0.2	0.1	Small	Small	Very Small
Recipes	NS	0.2	0.2	NS	Small	Small
Balanced Diet	0.2	0.2	0.1	Small	Small	Very Small
Paleo/HCG Diets	0.1	0.1	0.2	Very Small	Very Small	Small
Healthy Diet	0.1	0.2	0.2	Very Small	Small	Small
Physical Activity	0.3	0.4	0.4	Small	Small	Small
Mediterranean Diet	0.2	0.1	0.2	Small	Very Small	Small
Detox	0.1	0.2	0.1	Very Small	Small	Very Small
Ketogenic/LCHF Diets	0.1	0.2	0.3	Very Small	Small	Small
Obesity	0.1	0.1	0.2	Very Small	Very Small	Small
Celebrity Diets	NS	0.2	0.2	NS	Small	Small
Diet Change	0.1	NS	0.1	Very Small	NS	Very Small
Nutrient Information	NS	0.3	0.3	NS	Small	Small
No-Sugar Diet	0.2	0.1	0.3	Small	Very Small	Small
Atkins Diet	0.2	NS	0.2	Small	NS	Small
Healthy Diet Planning	NS	0.2	0.2	NS	Small	Small
Yo-Yo Dieting	0.2	0.1	0.1	Small	Very Small	Very Small
AAA Diet	NS	0.3	0.4	NS	Small	Small
Fitness Inspiration	NS	0.3	0.3	NS	Small	Small
Fitness Program	0.2	0.2	0.2	Small	Small	Small
Fitness Information	NS	0.2	0.1	NS	Small	Very Small
Dietary Log	NS	0.2	0.2	NS	Small	Small

**Table 4:** Top-10 high-weight topics of democratic, republican, and swing states.

Topics	Rank among Top-10 Topics			Class
	Dem (D)	Rep (R)	Swing (S)	
Self-Monitoring	-	8	9	RS
Weight Loss	4	-	-	D
Diet Information	-	-	10	S
Diabetes	9	-	-	D
Diet Sodas	7	1	1	DRS
Diet Promotion	-	-	7	S
Unhealthy Diet	8	2	2	DRS
Gluten-Free Diet	-	6	-	R
Balanced Diet	-	5	-	R
Healthy Diet	-	7	4	RS
Physical Activity	1	-	-	D
Mediterranean Diet	6	9	6	DRS
Nutrient Information	5	-	-	D
No-Sugar Diet	-	10	8	RS
AAA Diet	2	-	-	D
Fitness Inspiration	3	-	-	D
Fitness Program	10	3	5	DRS
Dietary Log	-	4	3	RS

**Table 5:** Categories of topics.

Category	Behavior and Lifestyle	Health Information	Chronic Condition	Type of Diet
Topics	Self-Monitoring Diet Sodas Unhealthy Diet Dietary Log Diet Promotion Physical Activity Healthy Diet Planning Fitness Inspiration Fitness Program Dietary Log	Weight Loss Diet Information Diet Pill Diet Education Recipes Healthy Diet Information Celebrity Diets Diet Change Nutrient Information No-Sugar Diet Fitness Information	Diabetes Obesity	Gluten-Free Diet Vegetarian/Vegan Diets Balanced Diet Paleo/HCG Diets Mediterranean Diet Detox Ketogenic/LCHF Diets Atkins Diet Yo-Yo Dieting AAA Diet

**Table 6:** Comparison of democratic (Dem), republican (Rep), and swing states. Not Significant (NS): adjusted *p*-value > 0.0005, significant (*): adjusted *p*-value ≤ 0.0005.

Category	ANOVA	Tukey Multiple Comparison Test		
	F-Value	Rep vs. Swing	Rep vs. Dem	Dem vs. Swing
Behavior and Lifestyle	28.1 *	* Rep > Swing	NS	* Dem > Swing
Health Information	453.1 *	* Rep < Swing	* Rep < Dem	* Dem > Swing
Chronic Condition	94.4 *	* Rep < Swing	* Rep < Dem	NS
Type of Diet	74.4 *	* Rep > Swing	* Rep > Dem	* Dem > Swing

**Table 7:** Cohen’s d and effect size of comparisons in Table 6.

Category	Mean of Cohen’s d of Sample Sizes			Effect Size		
	Rep vs. Swing	Rep vs. Dem	Dem vs. Swing	Rep vs. Swing	Rep vs. Dem	Dem vs. Swing
Behavior and Lifestyle	0.1	NS	0.1	Very Small	NS	Very Small
Health Information	0.2	0.2	0.2	Small	Small	Small
Chronic Condition	0.2	0.1	NS	Small	Very Small	NS
Type of Diet	0.3	0.1	0.1	Small	Very Small	Very Small

## Appendix A

**Table A1:** Definition of topics by independent reviewers after reviewing the top words per topic.

Topic	Description
Self-monitoring	The practice of observing health behaviors, actions, and decisions related to health.
Weight loss	Physical activity aimed at reducing weight and body mass index (BMI).
Diet Information	Information used to enable a person to take control over and improve their diet.
Diabetes	Chronic condition/disease in which blood glucose (sugar) levels are too high.
Diet Promotion and Advertisement	Information used to enable a person to take control over and improve their diet.
Diet Pill	Weight loss medications to reduce or control weight.
Unhealthy Diet	Food choices comprised of low nutritional value.
Gluten Free Diet	Dietary plan of eating foods that do not have gluten.
Vegetarian/Vegan Diet	Dietary plan abstaining from the consumption of meat and sometimes by-products of animal slaughter [75].
Diet Education	Information focused on increasing knowledge or spreading information related to Diet.
Recipes	Set of instructions for food preparation.
Balanced Diet	Fulfilling all of a person’s nutritional needs to function correctly.
Paleo/HCG Diet	While Paleo dietary plan is based on low-carbohydrate foods, the human chorionic gonadotropin (HCG) dietary plan is based on low-calories and low-fat foods [75].
Healthy Diet Information	Sharing information on food choices comprised of high nutritional value.
Physical Activity	Movement of the body by engaging in an organized or unorganized activity and exerting energy.
Mediterranean Diet	Dietary plan of primarily plant-based foods, healthy oils, and herbs and spices to flavor foods. Red meat is often limited to a few times a month or less [75].
Detox	A process or period of time in which a person abstains from or rids the body of toxic or unhealthy substances by flushing the system with water or naturally occurring products (fruits and vegetables).
Ketogenic/LCHF Diet	The low-carbohydrate, high-fat (LCHF) method is based on replacing carbohydrate with healthy fats. Consisting of low-carb foods forcing the body to turn fat into ketones for use as energy, Ketogenic diet is an example of LCHF diets [75].
Obesity	Having weight above what is considered healthy.
Celebrity Diets and Workouts	Physical activity routines promoted by celebrity social media accounts or associated with the celebrity name.
Diet Change	Changing eating habits.
Nutrition Information	Information on the nutrition value of a food.
No-Sugar Diet	Removing sources of added sugar from daily food intake.
Atkins Diet	A dietary pattern strict in low-carbohydrate foods [75].
Health Diet Plan	Develop a plan to consume food choices comprised of high nutritional value.
Yo-Yo Dieting	A process of losing weight, regaining it, and then dieting again.
Acid Alkaline Association (AAA) Diet	Dietary plan of eating 80% alkaline foods and 20% acid-producing foods [75].
Fitness Inspiration	Motivation to engage in fitness or become more active in lifestyle behaviors.
Fitness Program	Temporal representation of exercise or dieting behavior that is captured through specific constraints (e.g., minutes, day, morning, week).
Fitness Information	Information on the condition of being physically fit and healthy.
Dietary Log	Planning a consistent pattern of diets, such as cutting meat over time.
